# Supplementary material for: “When a man drinks alcohol it’s cool but when a woman drinks she is a hoe”: A qualitative exploration of alcohol, gender, stigma, and sexual assault in Moshi, Tanzania
Source: PLOS Glob Public Health. 2024 Feb 29;4(2):e0002382. doi: 10.1371/journal.pgph.0002382 (PMC10903863; doi:10.1371/journal.pgph.0002382)
Supplement: Attachment — Submitted filename: Response to Reviewers - Stigma.docx [file pgph.0002382.s003.docx]

January 16th, 2024

To Whom It May Concern:

We appreciate the helpful suggestions presented to us by our reviewers and thank them for the opportunity to improve our manuscript. We have incorporated the changes requested by both reviewers in our attached submission with our specific modifications outlined below. Our revised manuscript is attached with this resubmission packet.

Reviewer #1:

**Comment**: An interesting and well-written paper. This paper can give a new insight into understanding context like alcohol use from a gender lens, especially data collected through IDIs and analysis will help readers to understand the issue in depth. I would just suggest a minor revision that the IDI quotes need to be revised well. In some there are grammatical mistakes, which made meaning unclear.

*Response: We thank the reviewer for this kind assessment of our manuscript and the helpful feedback provided. We have fixed all grammatical mistakes within our in-depth interview quotes to better clarify the meaning.*

Reviewer #2:

I would like to express my gratitude to the editor for giving me the opportunity to review this manuscript, which I very much enjoyed reading. The authors present a study on a highly relevant, underresearched topic as part of a larger project of considerable importance. The title introduces the intricate interplay of alcohol use with dimensions on gender, stigma and violence, rendering the complexities of this massive global problem palpable through the voices of study participants. The manuscript is very well written. However, it has two major flaws that must be addressed, concerning its methodology and the discussion of findings.

**Comment**: Firstly, although the grounded theory approach is mentioned, there is a lack of appropriate reference and explanation. The description of methodology and findings raises doubts as to whether the authors have really applied grounded theory.

*Response: We thank the reviewer for bringing this gap to our attention. We have expanded the ‘Data Analysis’ section to better explain our use of grounded theory methodology in this manuscript.*

**Comment**: Secondly, the discussion is comprehensive and well written regarding the female angle, but it lacks depth as important findings are omitted. In particular, the absence of discussion on male behavior and societal perceptions is striking. A balanced examination of the implications, recommendations and conclusions for both genders and societal levels is warranted.

*Response: We thank the reviewer for this very valuable feedback and have made significant revisions to the discussion section in following with this and the following suggestions.*

In addition to that, I have minor comments and suggestions for the authors’ consideration to enhance the quality of this work.

**Comment**: Line 65, abstract: by reading the abstract I would expect a conclusion on both genders as the glorification of male drinking seems highly problematic and requires addressing

*Response: We have expanded our conclusion to incorporate a conclusion on male drinking rather than female drinking alone.*

**Comment**: Introduction: excellent, very clear and well written.

*Response: We thank the reader for this kind feedback.*

**Comment**: Line 109: stigma as an established barrier – needs a reference

*Response: We appreciate this comment from the reviewer. We have added citations on line 109.*

**Comment**: Line 134: IDI needs to be introduced, however, I suggest spelling out IDI throughout for better readability; the study already uses many abbreviations.

*Response: We thank the reviewer for this suggestion. We have changed all IDI abbreviations to their full, spelled-out form throughout the manuscript.*

**Comment**: Lines 154 ff: At this point, a section on reflexivity/positionality is needed in relation to the fact that the first author is not a local researcher and what led to this decision

*Response: We thank the reviewer for bringing this gap in reflexivity to our attention. To address this, we have added an additional paragraph to this section. This addition reads, “Of note, while this study was based in Moshi, Tanzania, the first author is not a local researcher. The local perspective has regardless been maintained in this paper as the Tanzanian research team informed all data collection tools and analysis procedures with the relevant sociocultural nuances of the study setting. The decision over authorship placement was made jointly between the Tanzanian and US research teams given that the first author spearheaded all study efforts starting from initial study development through data analysis and the subsequent formation of this manuscript.”*

**Comment**: Line 178: point 3) – how can perspectives and experiences be known before data collection?

*Response: We thank the reader for this question. To better clarify, we have added an explanation that these perspectives were first noted during survey collection to the end of this sentence.*

**Comment**: Line 192: include info on transcription

*Response: We appreciate this feedback from the reviewer. We have added greater detail on the transcription process, so that the following sentence now reads, “Directly after the initial collection, audio recordings were later translated transcribed into written KiSwahili and then translated into English by a member of the Tanzanian research team.”*

**Comment**: Line 206 ff: Please elaborate your step-wise approach with reference to key literature on grounded theory. There is insufficient description and, I’m afraid, probably also understanding of the grounded theory approach;

*Response: We appreciate this feedback from the reviewer and have made several adjustments to our manuscript accordingly. We have updated the section ‘Data Analysis’ to better detail our step-wise approach to analyzing the data using a grounded theory approach and have included more references used to appropriately guide this process.*

**Comment**: Line 239: Consider using ‘findings’ or ‘analysis’ rather than ‘results’ for qual research

*Response: We thank the reviewer for this feedback and have replaced the heading ‘Results’ with ‘Findings’ on line 239.*

**Comment**: Lines 246 ff: Nice summary of the findings, though not sure it is needed, consider removing if short on words

*Response: We thank the reviewer for this comment and suggestion. As we believe this summary helps to orient the reader to the findings that follow and there are no restrictions on word count for this journal, we have opted to keep this summary in the manuscript at this time.*

**Comment**: Line 255, table: the table refers to themes and sub-themes rather than providing a grounded theory analysis, probably due to an overall misunderstanding of the stated research approach

*Response: We appreciate this comment from the reader and acknowledge that differences in the presentation of a grounded theory analysis exist between various scholarly works. This format for Table 1 was specifically chosen after consulting with qualitative experts on our authorship team and reviewing several other grounded theory-based papers published in relevant, highly reputable journals that created similar tables for themes and subthemes. Table 1 is a direct product of our grounded theory methodology, presented to organize the various themes and subthemes that inductively emerged during data analysis and broadly outline our qualitative findings. As such, we maintain that this table is not a misunderstanding but rather a reflection of our stated research approach.*

**Comment**: Line 554 ff: The discussion focuses mainly on the impact on women and not only ignores the other side of the findings: the focus on women also risks looking at the phenomenon in isolation, whereas I am convinced that male, female and societal behavior and attitudes are interconnected and have to be discussed and addressed comprehensively. The study and its findings are too important for these aspects to be left out. I would like to see some discussion on, for example: Expectations are almost as if women had to behave like angels while being treated like inferior humans, slaves (sic); if their behaviour does not fully conform to societal norms, they are ousted

*Response: We appreciate the reviewer for bringing this gap in our discussion to our attention. We have incorporated this into our discussion. This addition now reads, “Compounding this division of labor, our analysis revealed an expectation for women to maintain constant, exemplary behavior and fulfill the role of a perfect wife and mother. Especially in the context of critical environments, quests for perfectionism and total infallibility have been linked with low self-esteem, low self-efficiency, anxiety, and depression… As explored more below, we found particularly severe consequences for women like complete social isolation when they failed to conform to societal expectations.”*

**Comment**: Women’s drinking behaviour is only a concern because it affects their labour force, society doesn’t seem to care about how alcohol affects women’s health and well-being

*Response: We thank the reviewer for this valuable feedback. We have expanded our discussion section to include a discussion of this point, which now reads, “Interestingly, participants mentioned concern around women’s drinking only as it would negatively affect their contribution to the labor force, but expressed no concern as to how it would affect their physical and mental health. This highlights an overlying societal view that the impact of alcohol use on labor was a prevailing concern among participants.”*

**Comment**: The same is true for men, except that in their case heavy drinking is either ignored or glorified, a hugely damaging trivialization at the detriment of men’s health

*Response: We appreciate this helpful suggestion from the reviewer. We have expanded the discussion section to allow for a thorough review of this point. Our addition now reads, “In contrast to the disproportionate stigmatization of women’s drinking, men received little to no public censure for their alcohol use, even when obviously problematic. Public reproval was noted to fall on their families, while the men themselves were embraced and even admired for their drinking, a damaging trivialization of problematic behavior that could drive excessive consumption. In Moshi, men are known to drink at higher rates than women and are more likely to be diagnosed with alcohol use disorder and experience alcohol-related injuries. Societal encouragement of men’s alcohol consumption could contribute to these high rates of use and negative impacts on men’s health. Previous work by our team has identified that in Moshi, social pressures contribute to men’s drinking as their social interactions with other men typically occur in bars or other locations where drinking is encouraged. As such, future efforts to minimize men’s alcohol use should be considerate of social pressures to drink and incorporate provisions to address this important factor.”*

**Comment**: Thorough elaboration on the problematic behaviour of men, both in terms of their alcohol consumption and their use of alcohol to justify and perpetrate SGBV against alcoholised women

*Response: We appreciate this suggestion from the reviewer and have revised our manuscript accordingly.*

**Comment**: eg. line 618: alcohol use facilitate this behaviour, but also the lack of sanctions/punishment => programmes must focus on support of affected women plus legal consequences for men plus changing societal attitudes

*Response: We thank the reviewer for bringing this important insight to our attention and have adjusted our manuscript accordingly. Alongside some changes in phrasing that highlight the lack of legal repercussions for perpetrators of sexual violence, our discussion section is now concluded with the following sentences. “Given the notable harm identified by our study participants, we recommend future researchers and policymakers work to develop interventions to fit the sociocultural context of Moshi and implement them accordingly. This, in addition to addressing the lack of legal repercussions for perpetrators of sexual violence and raising societal awareness around these issues, will ensure greater support for and protection of the Moshi community.”*

**Comment**: Programmes to mitigate this problem need to provide solutions for both genders and include awareness raising measures at a societal level

*Response: We appreciate this comment from the reviewer and have incorporated this addition in the second to last paragraph of our discussion section.*

**Comment**: Line 649: can also be seen as a strength that – although not explicitly asked - the data collection process allowed this important topic to emerge

*Response: We thank the reviewer for this suggestion. We have rephrased this sentence of the limitation section, which now reads, “Another limitation is that questions specifically pertaining to gender roles and sexual assault were not included in the interview guide, which means that some perspectives may not have been included from interviewees. This omission, however, also highlights the strength of our data collection process in allowing important topics to emerge organically and showcases how significant gender roles and sexual assault are in the context of local alcohol use.”*

**Comment**: Lines 660 ff: adjust conclusion taking into account the above points

*Response: We appreciate this contribution from the reviewer and have adjusted the conclusion accordingly.*
